# From Residues to Added-Value Bacterial Biopolymers as Nanomaterials for Biomedical Applications

**DOI:** 10.3390/nano11061492

**Published:** 2021-06-04

**Authors:** Francisco G. Blanco, Natalia Hernández, Virginia Rivero-Buceta, Beatriz Maestro, Jesús M. Sanz, Aránzazu Mato, Ana M. Hernández-Arriaga, M. Auxiliadora Prieto

**Affiliations:** 1 Interdisciplinary Platform for Sustainable Plastics towards a Circular Economy-Spanish National Research Council (SusPlast-CSIC), 28040 Madrid, Spain; francisco.blanco@cib.csic.es (F.G.B.); nhernandezh@cib.csic.es (N.H.); mvrivero@cib.csic.es (V.R.-B.); aranzazu.mato@gmail.com (A.M.); arriaga@cib.csic.es (A.M.H.-A.); 2 Polymer Biotechnology Group, Microbial and Plant Biotechnology Department, Biological Research Centre Margarita Salas, CIB-CSIC, 28040 Madrid, Spain; 3 Host-Parasite Interplay in Pneumococcal Infection Group, Microbial and Plant Biotechnology Department, Biological Research Centre Margarita Salas, CIB-CSIC, 28040 Madrid, Spain; beatriz.maestro@cib.csic.es (B.M.); jmsanz@cib.csic.es (J.M.S.)

**Keywords:** bacterial polymers, bacterial cellulose, polyhydroxyalkanoates, γ-polyglutamic acid, upcycled polymers, biomedical applications, biopolymer functionalization

## Abstract

Bacterial biopolymers are naturally occurring materials comprising a wide range of molecules with diverse chemical structures that can be produced from renewable sources following the principles of the circular economy. Over the last decades, they have gained substantial interest in the biomedical field as drug nanocarriers, implantable material coatings, and tissue-regeneration scaffolds or membranes due to their inherent biocompatibility, biodegradability into nonhazardous disintegration products, and their mechanical properties, which are similar to those of human tissues. The present review focuses upon three technologically advanced bacterial biopolymers, namely, bacterial cellulose (BC), polyhydroxyalkanoates (PHA), and γ-polyglutamic acid (PGA), as models of different carbon-backbone structures (polysaccharides, polyesters, and polyamides) produced by bacteria that are suitable for biomedical applications in nanoscale systems. This selection models evidence of the wide versatility of microorganisms to generate biopolymers by diverse metabolic strategies. We highlight the suitability for applied sustainable bioprocesses for the production of BC, PHA, and PGA based on renewable carbon sources and the singularity of each process driven by bacterial machinery. The inherent properties of each polymer can be fine-tuned by means of chemical and biotechnological approaches, such as metabolic engineering and peptide functionalization, to further expand their structural diversity and their applicability as nanomaterials in biomedicine.

## 1. Introduction

Ongoing global population growth and aging imply an increase in global demand for sustainable development, which involves the rational use of resources and the maintenance of ecosystem services [1]. This calls for more efficient production methods in order to render industrial and technological development compatible with social wellbeing and environmental protection. Consequently, there is a need for environmentally friendly and low-impact methodologies in manufacturing processes, aimed at reducing byproducts whilst upcycling waste [2]. In this sense, increasing pressure on the environment due to the widespread consumption of petroleum-based polymers has hastened the development of biodegradable and environmentally friendly materials such as bio-based polymers. Biopolymers are naturally occurring materials comprising a wide range of molecules with diverse chemical structures that can be produced in a sustainable manner from renewable sources, in compliance with the United Nations Sustainable Development Goals and the concept of the circular economy.

Biopolymers are widely applied for biomedical purposes since they are generally biocompatible and biodegradable into nontoxic products; moreover, they present low antigenicity and high bioactivity, they can be processed into complicated shapes, they are capable of supporting cell growth and proliferation, and they exhibit highly diverse thermal and mechanical properties [3]. These inherent properties can be fine-tuned by means of biotechnological and chemical approaches. Current strategies based on cutting-edge technologies, such as synthetic and systems biology combined with advanced materials technology, provide pathways for enhancing the structural and functional complexity of these biopolymers, thereby expanding the catalog of available biomaterials beyond that which exists in nature and extending their potential applications in the biomedical sector (e.g., drug delivery, tissue engineering) [3]. The bottom-up strategy of material design opens up important opportunities for the creation of specific cutting-edge biomedical applications [4].

In particular, bacterial polymers have attracted much attention over the last decade due to their sustainable production and the fact that their properties can be altered with the use of bioengineering tools. In the context of the circular economy, bacteria are able to grow and produce materials of interest from complex carbon sources such as industrial and municipal wastes. It entails developing bioprocesses in order to upcycle the abovementioned waste into added-value materials with application in numerous industrial sectors. Indeed, bacteria produce a broad range of polymers as part of their inherent physiology, and many of these are currently being used as materials for biomedical applications [5]. Some examples are polysaccharides, including alginates, hyaluronic acid, and bacterial cellulose (BC); polyesters, comprising the family of polyhydroxyalkanoates (PHAs); polyamides, which are amino acid polymers synthesized in a ribosome-independent manner, such as cyanophycins, γ-polyglutamic acid (PGA), or poly-Ɛ-lysine; and, finally, polyanhydric polymers, such as polyphosphates, that are produced by a wide variety of bacteria for use as energy storage polymers [5].

Bacterial polymers have grown exponentially in biomedical research, mainly in three domains: drug nanocarriers, implantable material coatings, and tissue-regeneration scaffolds or membranes. Different types of nanocarriers loaded with a particular drug (namely, nanospheres, nanocapsules, polymeric micelles) are employed for drug delivery because they enhance the pharmacokinetic and pharmacodynamic profile of the drugs by increasing the bioavailability of bioactive molecules that present poor solubility in water, promoting sustained release and enhancing permeability across biological barriers. Furthermore, they can reduce side effects by enabling targeted and controlled drug release [6]. However, these nanocarriers interact massively with their environment, e.g., biological fluids and cells, where they are rapidly removed by the mononuclear phagocyte system. In order to prolong their half-life, surface modification by means of coating with biopolymers (or direct formulations of biopolymer nanocarriers) has largely proven to confer stealth properties to the resulting nanosystems [7], which help to evade the immune system, thus prolonging their therapeutic effects. Bacterial polymers are particularly interesting for nanocarrier formulations due to their intrinsic biocompatibility and biodegradability properties, which enable the release of the encapsulated compound associated with the degradation of the polymeric matrix into nontoxic monomers.

Another area of interest in relation to biopolymers involves surface coatings of temporary or permanent implantable medical devices. The main issue associated with implant failure continues to be associated with bacterial adhesion and subsequent biofilm formation on the device surface. Different strategies have been employed to prevent colonization by bacteria, such as the design of nanostructured antibacterial topologies [8], surface coating of the implant with intrinsic antimicrobial polymers [9], chemical modification of surface materials to prevent adhesion or to provide antibacterial activity [10], and the immobilization of antimicrobial peptides, enzymes, or inorganic compounds [11]. Due to the emergence of antibiotic-resistant bacteria, the use of functional nanomaterials to control device-associated infections has been proposed as a promising alternative to conventional antibiotic treatment [12].

Finally, bacterial polymers present a series of advantages in the fields of tissue regeneration and wound healing. When formulated as hydrogels, membranes, or 3D scaffolds, due their highly swollen three-dimensional environment, bacterial polymers can simulate an extracellular cell matrix (ECM) structure, providing the damaged tissue with a friendly environment for regeneration [13]. Furthermore, many strategies have been reported to functionalize these materials with cell-attachment motives, antimicrobial functionalities, or specific cell type effectors (i.e., growth factors, cytokines) with the aim of enhancing and accelerating tissue regeneration and wound closure [13].

**Figure 1:**
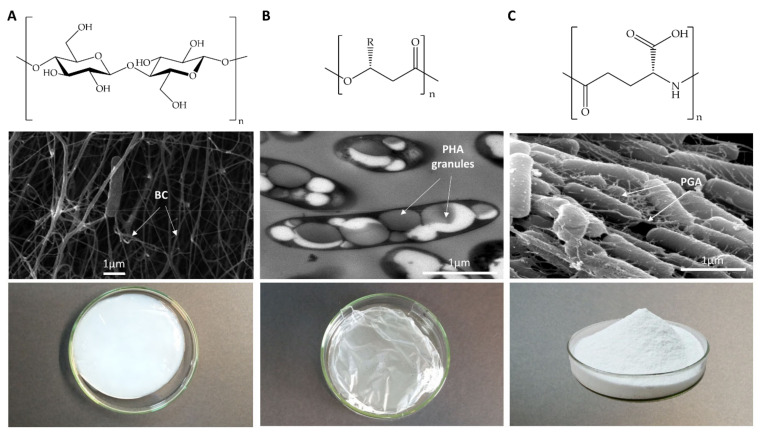
Chemical, microscopic, and macroscopic structure of BC, PHA, and PGA produced by model bacteria *Komagataeibacter medellinensis*, *Pseudomonas putida*, and *Bacillus subtilis*. Upper panels represent the chemical polymer structure, middle panels show electron microscopy images of the microorganisms producing the polymer, and lower panels show the macroscopic appearance of the purified polymer of BC (**A**), PHA (**B**), or PGA (**C**). SEM images of *K. medellinensis* and *B. subtilis*. Reprinted with permission from [14,15]; Copyright Microbiology Society, 2013, 2006.

The present paper focuses on three bacterial biopolymers (one of each carbon backbone structure) suitable for biomedical applications in nanoscale systems. In particular, we focused upon the three most technologically advanced ones (PHA, BC, and PGA). Herein, they are considered to constitute models of each carbon-backbone structure (polysaccharides, polyesters, and polyamides) (Figure 1 and Table 1). We will emphasize their suitability for applied sustainable bioprocesses based on renewable carbon sources, their modification to further expand their structural diversity, and their applicability for biomedical purposes.

## 2. BC, PHAs, and PGA as Model Bacterial Biopolymers Produced by Upcycling Bioprocesses

Bacteria exhibit wide-ranging metabolic versatility, which is also reflected by the great variety of polymers they are able to naturally synthesize. Polymeric matrices are involved in diverse cell functions, such as promoters of bacterial adhesion, energy and carbon storage, pathogenicity factors, or biofilm constituents [5].

### 2.1. Molecular Basis of the Biopolymer Synthesis

**Figure 2:**
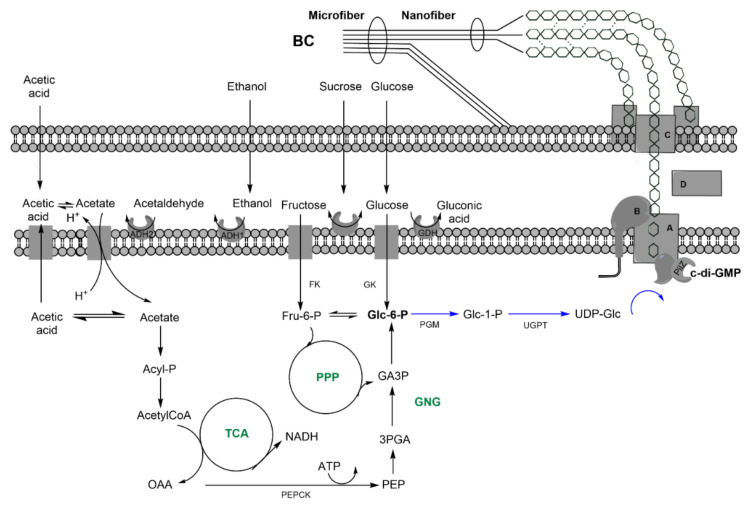
Metabolic network of BC in *Komagataeibacter xilynus* E25. Sugars are metabolized through the pentoses phosphate pathway (PPP) to Glc-6-P, while glycolysis is not a relevant pathway in *Komagataeibacter* species due to the lack of phosphofructokinase. Glucose is partially oxidized in the periplasm to obtain reductor power. In the case of *K. xylinus* E25, the oxidation product is gluconic acid, although the final product is species-dependent. Ethanol is dehydrogenized to acetate by ADH1, an ADH2 inner-membrane-bound enzyme, and directed to Glc-6-P by the tricarboxylic acid cycle (TCA) and gluconeogenesis (GNG) pathways. Glc-6-P is isomerized to Glc-1-P by phosphoglucomutase (PGM) and is subsequently transformed to UDP-Glc by UTP–Glc-1-P uridylyltransferase (UGPT). Upon activation by c-di-GMP of BcsA, UDP-Glc units are polymerized into nascent glucan chains coupled with its translocation to the periplasm by means of cellulose synthase subunits BcsA (A) and BcsB (B). BcsC (C) is then involved in the arrangement of the nascent chains, and BcsD (D) forms the pore to export the nanofibrils. The main metabolic pathways, TCA, GNG, and PPP, are indicated in green. The pathway leading to BC synthesis is indicated in blue. Key enzymes, phosphoglucoisomerase (PGI), phosphoenol pyruvate carboxykinase (PEPCK), PGM, and UGPT are indicated. OAA: oxalacetate; PEP: phosphoenol pyruvate; 3PGA: 3-phosphoglycerate; GA3P: glyceraldehyde-3-P.

BC is a linear, extracellular polysaccharide composed of chains of β(1,4) O-glycosidic-bounded glucose (glc) units (Figure 1A). These chains aggregate upon extrusion into nanofibers to further form a 3D-structured microfiber network (Figure 2).

It is produced by bacteria of the genera *Acetobacter, Agrobacterium, Azotobater, Pseudomonas, Rhizobium, Escherichia, Salmonella,* or *Komagataeibacter* [16]. However, the species of the *Komagataeibacter* genus are the most effective nanocrystalline BC producers. *K. xylinus* is the model organism that is most industrially exploited. This bacterium has undergone various phylogenetic reclassifications over the years, originally named *Acetobacter xylinum*, subsequently *Gluconacetobacter xylinum*, and finally renamed as *Komagataeibacter xylinus* [17]. To avoid any misunderstanding, our paper will refer to these strains as they were named in the original article. The proposed polymer function varies from one species to another; for example, these functions can involve protection against ultraviolet radiation and moisture retention, retaining the bacteria in an aerobic environment, or enhancing surface colonization [18].

BC is produced by the polymerization of units of 1-uridine diphosphate glucose (1-UDP-glc) (Figure 2). Glucose is first phosphorylated to glucose-6P, isomerizated to glc-1P, and finally converted into 1-UDP-glc [19]. The monomeric unit, UDP-glc, is polymerized and translocated by cellulose synthases BcsA and BcsB (or the fused version, BcsAB) in a process regulated by BcsA allosteric activator cyclic di-guanosine monophosphate (c-di-GMP) [20]. BcsC constitutes an outer membrane translocase, while periplasmic nonessential BcsD is involved in the fibrils’ hierarchical arrangement [18]. These four genes, *bcsABCD,* form the cellulose synthase operon. Depending on the operon type and species, accessory genes modulating BC synthesis may be present, such as *bcsZ* (endo-β-1,4-glucanase), *ccpAx*, or *bglAx* (β-glucosidase) [18,21].

The preference for one carbon source or another is strain-dependent [22] as a result of the genome flexibility of the genus [23]. Disaccharides, such as sucrose, are hydrolyzed in the periplasm, and the monomers are actively translocated in phosphorylation [24]. Glucose-6P can either be converted into the BC precursor, 1-UDP-glc, or further catabolized by central carbon pathways. Most of the *Komagataeibacter* genus lack or present very low phosphofructokinase activity; hence, the Emden-Meryorf-Parnas pathway is not present in *K. xylinus*. Subsequently, glucose is metabolized by partial oxidation to gluconate-6P or by decarboxylation into ribulose-5P, entering the pentoses phosphate pathway (PPP). Another BC precursor is ethanol, which can be dehydrogenated into acetate by the membrane-bound alcohol and aldehyde dehydrogenases (ADH1 and ADH) (Figure 2) and is incorporated as acetate into the cell. Further phosphorylation results in acetyl-CoA, which feeds a glyoxylate-shunted tricarboxylic acid (TCA) cycle, from which oxalacetate can be decarboxylated to render phosphoenol pyruvate, the starting point of gluconeogenesis (GNG), which, in turn, feeds the glc-6P pool [25,26]. Furthermore, ethanol has been reported as a BC production stimulant via upregulation of UDP-glc- and *bcs*-related genes. Moreover, it downregulates those of the IS110 transposase family (involved in the appearance of celluloseless phenotypes) and synthesis genes associated with byproducts [27]. These byproducts, mainly water-soluble exopolysaccharides (EPSs) such as acetan, are strain-dependent, and the effect of their synthesis upon BC yield remains unclear. Other BC carbohydrate precursors such as galactose or xylose have been described in the literature (see below).

**Figure 3:**
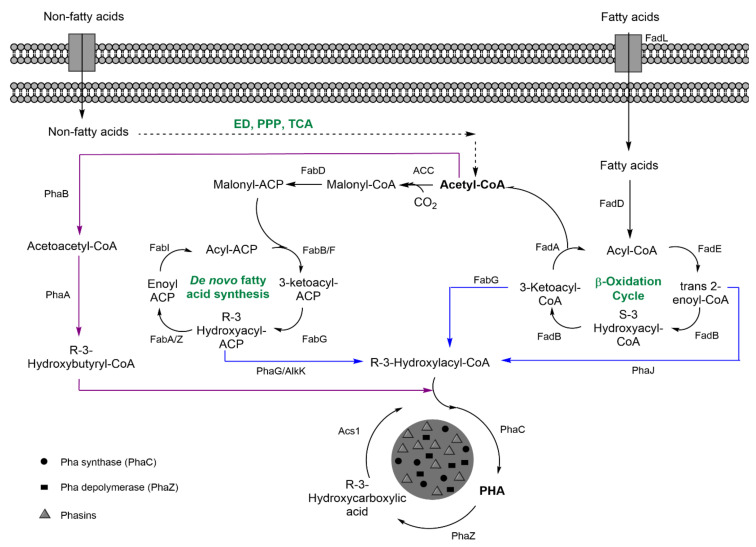
Metabolic network of PHA metabolism in model bacteria *C. necator* H16 (scl-PHA) and *P. putida* KT2440 (mcl-PHA). Fatty acids are metabolized via the β-oxidation cycle into acetyl-CoA, while nonfatty acid substrates are metabolized via the Entner-Doudoroff (ED) pathway, TCA and PPP, into acetyl-CoA. In PHB metabolism, two acetyl-CoA molecules condensate into acetoacetyl-CoA by PhaB and are then converted into *R*-3-hydroxybutyryl-CoA (PHB monomer) by PhaA. In PHA metabolism, fatty acids are metabolized in the β-oxidation cycle, where the intermediates 3-ketoacyl-CoA and trans-2-enoyl-CoA can be directly converted into PHA monomers (R-3-HA-CoA) by FabG and PhaJ, respectively. Alternatively, through de novo fatty acid synthesis, acetyl-CoA can be converted from R-3-hydroxyl-ACP to R-3-HA-CoA by two enzymatic steps catalyzed by PhaG and AlkK. PHA and PHB are synthetized in a continuous cycle that drives carbon and energy flux, in which the monomers are polymerized by PhaC, depolymerized into the respective *R*-3-hydroxycarboxylic acids by PhaZ, and reconverted into the activated monomer R-3-HA-CoA by Acs1. The carbon central metabolic pathways are indicated in green, the pathway leading to PHB synthesis is in purple, and the one leading to PHA synthesis is shown in blue. Key enzymes are indicated.

PHAs are linear, intracellular polyesters of R-3-hydroxyalkanoate units (Figure 1B) that accumulate in the cytoplasm as hydrophobic inclusions or granules (100–500 nm) coated with a series of granule-associated proteins (GAPs) involved in PHA metabolism and granule formation (Figure 3). These proteins include polymer synthases, depolymerases, and phasins, the latter constituting the major fraction of GAPs that isolate the hydrophobic PHA granule from the hydrophilic cytoplasm and ensure granule number and size, in addition to other physiologic functions [28]. PHAs are produced upon nutrient imbalance as a carbon and energy storage, and the PHA cycle regulates carbon flow in the cell [29]. To date, over 90 genera, including photoautotrophs and chemotrophs, Gram-positive and Gram-negative genera, are known to produce PHA under both aerobic and anaerobic conditions [30]. Furthermore, several recombinant strains have been developed to produce PHA. Despite the fact that the most common substituent groups found in PHA monomers are aliphatic chains, over 150 different monomer constituents have been described [31]. They are often classified as short-chain-length PHA (scl-PHA) or medium-chain-length PHA (mcl-PHA) based on monomer size, with 3–5 or 6–14 carbon units, respectively. The type of PHA synthesized depends upon the type of PHA synthase (PhaC), which can accept precursors of a certain carbon length, as well as the substrate employed and the metabolic and regulatory networks in each species [31].

Figure 3 summarizes the PHA metabolism for two model scl- and mcl-PHA producers, *C. necator* and *P. putida*, respectively. Scl-PHAs, namely, polyhydroxybutyrate (P(3HB)), are synthesized by condensation of two molecules of acetyl-CoA to acetoacetyl-CoA by PhaA, a β-ketothiolase, and reduced to 3-hydroxybutyryl-CoA, the PHB monomer, by acetoacetyl-CoA reductase PhaB. The monomers are then polymerized by PhaC synthase. Mcl-PHA structures are more diverse since they are mainly produced as heteropolymers of R-3-hydroxyacyl-CoA (R-3-HA-CoA) monomers of different lengths. Monomers are provided as intermediates of fatty acid metabolism via the β-oxidation pathway and de novo fatty acid synthesis. β-oxidation degrades fatty acids and provides trans-Δ2-enoyl-CoA, S-3-hydroxyacyl-CoA, and 3-ketoacyl-CoA metabolites that can be converted into R-3-HA-CoA by enoyl-CoA hydratase PhaJ, 3-HA-CoA dehydratase/epimerase FadB, and 3-ketoacyl-CoA reductase FabG, respectively. De novo fatty acid synthesis incorporates indirect precursors such as carbohydrates through their complete oxidation to acetyl-CoA and conversion via the TCA cycle into malonyl-CoA [29,30]. The latter CoA group is replaced by an acyl carrier protein (ACP), and the malonyl-ACP is further elongated by the addition of successive acetyl-CoA units. The resulting R-3-hydroxyacyl-ACP intermediates can be converted to R-3-HA-CoA monomers by PhaG thioesterase and the fatty acid CoA ligase (alk1), thus producing the substrates of PhaC [32]. PHA metabolism is involved in a continuous cycle of synthesis (PhaC) and degradation (PhaZ), determining PHA turnover in which the monomers can be metabolized via β-oxidation or reincorporated into the granule [33].

PGA is a linear, water-soluble, extracellular polyamide consisting of L, D, or both enantiomers of glutamic acid (glu). The units are linked by an amide bond between the amino and γ-carboxy groups of the glu residues (Figure 1C). The physiological function of PGA has not yet been completely elucidated, although it is believed to depend on the microorganism environment and whether it is bound to peptidoglycan [34]. Phylogenetically, two PGA producer groups can be distinguished: non-*Bacillus* (i.e., *Planococcus halophilus* or *S. epidermidis*) and *Bacillus* species. Some of the bacillary species can synthesize PGA attached to the peptidoglycan layer (i.e., *B. anthracis*), whereas others release it into the medium. The latter group, which includes *B. subtilis*, *B. licheniformis,* and *B. amyloliquefaciens*, together with glu-independent producers (that produce glu monomers through de novo synthesis), is the principal species exploited for the industrial production of PGA [35,36]. However, the low level of productivity of glu-independent bacteria has led to the development of genetically modified strains presenting a heterologous glu biosynthetic pathway [37,38]. In recent years, different heterologous hosts have been postulated as effective PGA producers, including *Escherichia coli* and *Corynebacterium glutamicum* [39,40].

**Figure 4:**
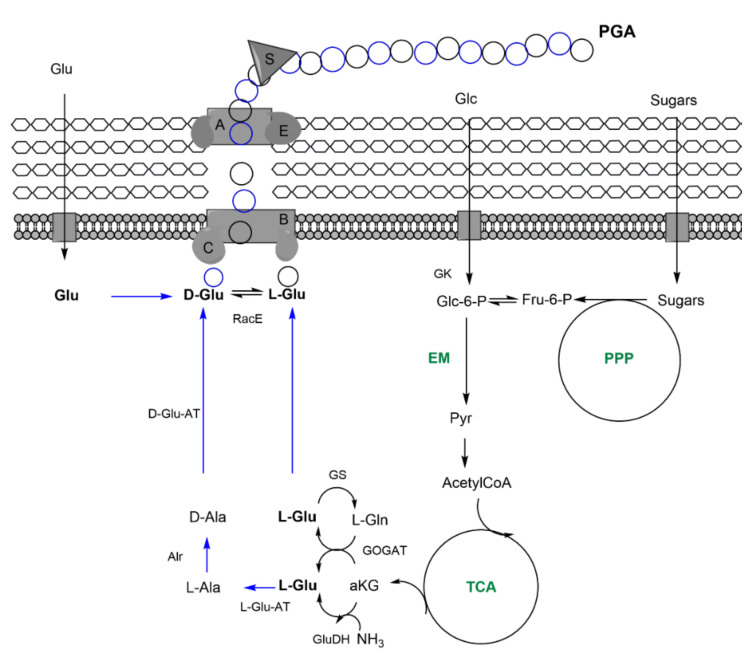
Metabolic network of PGA metabolism in model bacteria *B. subtilis* 168. Exogenous glu can serve as a direct precursor of PGA synthesis. Alternatively, sugars are metabolized via the Embden Meyerhof (EM) pathway, PPP and TCA. αKG from TCA is then converted to L-glu (the PGA monomer) by two different enzymes—glutamine oxoglutarate aminotransferase (GOGAT), which transfers the amino group from a glutamine molecule, and Glu dehydrogenase (GluDH), which incorporates the amino group from an ammonium molecule. In the species producing L/D-PGA (such as *B. subtilis*), a racemization reaction to produce the D-isomer takes place. This can be produced by means of two different enzymatic reactions: the Glu racemase (RacE) directly interconverting the isomers and a 3-enzymatic-step reaction by L-glu-pyruvate aminotrasnferase (L-Glu-AT), alanine racemase (Alr), and D-glu-pyruvate aminotransferase (D-Glu-AT). D/L glu monomers are then polymerized in the active site formed by membrane-bound PgsB (B) and PgsC (C), and the elongated chain is then removed from the active site by PgsA (A). The role of PgsE (E) is still under debate, while PgdS (S) is a secreted peptidase that releases the PGA to the medium. The main metabolic pathways, TCA, GNG, and PPP, are indicated in green. The pathway leading to PGA synthesis is indicated in blue. Key enzymes, glucokinase (GK), GOGAT, GluDH, glutamine synthetase (GS), L-Glu-AT, Alr, D-Glu-AT, and RacE are indicated.

The molecular machinery involved in PGA synthesis is organized in *B. subtilis* in an operon containing the synthase complex PgsBCA, the downstream gene *pgsE*, and the peptidase PgdS [41]. PgsB and PgsC form the active site of the synthase complex, in which ATP binds the γ-carboxyl group of a glu residue and is then eliminated by the amino group of another glu molecule by means of a nucleophilic attack, resulting in an n+1 chain. PgsA then becomes involved in removing the elongated chain from the active site in order to enable another monomer to be added. The role of *pgsE*, located downstream of *pgsBCA,* is still under debate [36]. The *pgsBCA* operon is controlled by both intracellular DegS/DegU two-component and quorum-sensing systems [42]. Finally, PgdS is an endoamydase involved in PGA chain cleavage and the release of the PGA chain into the medium (Figure 4).

The preference for carbon source is strain-dependent, with glucose generally favoring PGA production [35]. The endogenous production of glu is driven by carbon catabolism through the TCA cycle, from which the α-KG intermediate serves as a direct precursor of glu in a reaction mediated by glu dehydrogenase (GDH). Glu can also be obtained from the glu-glutamine cycle by the transference of the amino group from glutamine to α-KG by glutamate synthase (GOGAT). Conversely, exogenously provided glu can be directly included in PGA growing chains. Conversion of L-glu to its D enantiomer, necessary for some Bacillus’ peptidoglycan, as well as for those species producing D-PGA or D/L-PGA, is catalyzed by glu racemases (*racE/glr* and *yrpC* in *B. subtilis*) or in an indirect reaction by means of 3 enzymes (L-glu-pyruvic acid aminotransferase, L-alanine racemase, and D-glu-pyruvic acid aminotransferase) (Figure 4) [34]. Importantly, many *Bacillus* species produce different EPSs that, besides diverting resources from PGA production, hinder PGA recovery and purification [43].

### 2.2. Main Properties of Natural Model Biopolymers

Bacterial biopolymers have found their place in the biomedical field due to the properties they possess: (i) Most of them are biodegradable within the human body and, furthermore, their degradation rate can be tuned to meet the required shelf-life, whereas nondegradable polymers can serve as permanent implant materials; (ii) they are biocompatible, and their degradation compounds are nontoxic, nonimmunogenic, and noncarcinogenic; and (iii) they present adequate and tailorable mechanical and thermal properties for different applications. In this section, we focus on the properties of raw PHA, BC, and PGA, and, in Section 5, we analyze the possibilities of tuning their synthesis in order to generate specific properties.

One of the most valuable properties of bacterial polymers involves their biodegradability. Although BC, PHA, and PGA are degradable in nature, BC is not degraded in the human body due to the lack of efficient hydrolytic enzymes. Spontaneous degradation, which is still under debate, is possibly due to unspecific slow chemical hydrolysis, which makes BC a suitable material for long-term implants. However, attempts have been made to tune the degradability of this polymer (i.e., degradable NAcGlc-modified BC [44] or cellulase-conjugated BC; see Section 5 for more details). Conversely, both PHA and PGA are biodegradable in the human body. PHA is susceptible to nonspecific enzymatic degradation (i.e., lipases and esterases) [45]. Degradation rates strongly depend upon the enzymatic microenvironment of the target tissue as well as on monomer composition (e.g., length of the chain, presence of functional groups). Likewise, PGA has been reported to be rapidly hydrolyzed in vitro under acidic (pH 4) or basic conditions (pH 9) [46], proving to be rather stable under neutral conditions (pH 7). In addition, different enzymes can act on the peptide bonds, leading to subsequent degradation. These enzymes include human proteases such as trypsin and pronase E [47]. However, in vivo biodegradability of this polymer is still under research and appears to require a rather acidic environment [48].

Biocompatibility involves eliciting a proper host response upon implantation; it refers to the absence of cytotoxicity, genotoxicity, hemolysis, carcinogenicity, oxidative stress, or immune response, among others. Since biopolymers are degraded into common cellular building blocks, such as sugars, amino acids, or fatty acids, they are generally biocompatible. BC and PHA have proven to be biocompatible in multiple in vitro and in vivo studies (hemocompatibility, cell attachment, proliferation, toxicity, and metabolic assays) [49,50], although attention must be paid to the purification process to ensure endotoxin removal when producing PHA in Gram-negative strains. Furthermore, its degradation products—R-3-hydroxyacids—are nontoxic to the human body and have a much weaker impact on local pH than their synthetic counterparts (i.e., poly lactic acid). For instance, 3-hydroxybutyrate, a degradation product of P(3HB), is a natural component of human blood. Similarly, PGA and its degradation compounds L- and D-glu have been extensively proven to be biocompatible in vitro [51] and in vivo [52].

Material chemical and mechanical properties are key parameters that determine the feasibility of manipulation and molding. Additionally, with regard to application, they create the proper microenvironment for cell growth, which is known to be driven by stiffness sensing [53], and determine water availability (hydrophilicity and hygroscopic properties). We subsequently highlight the material properties of raw BC. Although PHA and PGA can be employed directly in some applications, they are more likely to be used after chemical modification or mixed with other materials, a fact that can greatly affect the properties described in the following paragraphs.

BC, which is naturally synthesized as a hydrogel, possesses a Young’s modulus (*E*) of 15–30 GPa, average tensile strength (σ_t_) of 250 MPa, and an elongation at break (ε_b_) of 10% [54]. These parameters are in the order of the values for soft tissues. Nonetheless, it is interesting to note that the values vary greatly from one strain to another and according to culture conditions. Unlike plant cellulose, BC can be molded upon production; it is synthesized chemically pure, and its degree of polymerization is higher than that of plant cellulose (800–10,000 vs. 700–1400) [55]. The 3D network structure of BC exhibits a high degree of crystallinity (60–80%), which accounts for its thermal stability [56]. Moreover, its structure confers on BC a high aspect ratio, resulting in a large surface area, with free hydroxyl groups that provide BC with good water-holding capacity (WHC), up to 99% of its total weight [56]. Dehydrated BC is rarely applied in the biomedical field. BC subjected to thermal drying displays 14 times less WHC than in its native (hydrated) form. Additionally, a significantly higher rate of deformability (2.7-fold, measured as ε_b_) was found for the hydrated form than for dry BC. Conversely, Young’s modulus and σ_t_ were found to be higher for the dried form [57]. These parameters are summarized in Table 2.

PHAs constitute a whole family, and their mechanical and thermal properties vary greatly according to the composition of the monomer and the molecular weight of the polymer. Scl-PHAs are highly crystalline, brittle, and stiff [65], whereas mcl-PHAs are thermoplastic elastomers presenting a high degree of elasticity; both polymers exhibit low oxygen permeability and high water resistance [65,66]. For instance, P(3HB) presents an *E* of 3.5 GPa, a σ_t_ of 40 MPa, and an ε_b_ of 6%, while mcl-PHA poly(3-hydroxydecanoate) (P(3HD)) presents an *E* of 19.8 MPa, a σ_t_ of 12 MPa, and an ε_b_ of ca. 300% [67]. Some of these properties are summarized in Table 3 for various PHAs and will determine the target applications of the material.

Furthermore, PGA is a water-soluble anionic polymer when it forms salts with cations, whereas its free acid form is insoluble in water. The conformation and viscosity of PGA in solution is pH-dependent, which calls for the creation of pH-responsive materials. PGA in its acid form presents a rod-like shape with an α-helix secondary structure, whereas Na-PGA folds preferentially into random coils, acquiring a sphere-like conformation [80]. As a raw biomaterial, its principal properties involve its highly hygroscopic and moisturizing effects, comparable to those of hyaluronic acid.

## 3. Sustainable Production of Bacterial Polymers

The three model biopolymers addressed in the present paper are produced by means of biotechnological processes based on different strategies in terms of bioprocess engineering. However, all three are produced from renewable sources such as waste streams. This section first provides a general description of each bioprocess, subsequently giving an overview of their production from different feedstocks.

### 3.1. General Aspects of the Bioprocess

BC synthesis by the *Komagataeibacter* genus is characterized by its low titer, yield, and productivity. This is mainly due to two essential features of the process. On the one hand, production is coupled to growth, with production strains presenting low growth rates. On the other hand, production mainly relies on static culture strategies to avoid the induction of B- defective phenotypes, which reduces BC yield and alters its mechanical properties [81]. Under static conditions, oxygen is less available and growth is diminished, but the downstream process is facilitated; this consists of removing the membrane from the air-medium interface, boiling it with NaOH, and rinsing it with water until the pH becomes neutral [82]. Although there is abundant literature on the development of bioreactors, which has been revised elsewhere [83], configurations preventing the loss of mechanical properties and providing high titer production have not yet been achieved. Due to the scant arsenal of available molecular biology techniques applied to BC-producing bacteria, attempts to enhance BC production are predominantly based on the replacement of the culture medium or carbon source with wastes as an alternative feedstock [62,84,85].

The operational mode for the industrial production of PHAs depends on the producing strain selected. Traditional production relies on pure culture strategies, in which PHA accumulation is initiated upon nutrient imbalance. This production strategy is oriented towards high cell density cultivation. Although batch productions are also used, fed-batch strategies have been shown to provide the highest PHA production yields. Fed-batch cultures are fed with nutrient sources, enabling periods of growth and PHA accumulation, thus maximizing cell growth [86]. Alternative strategies, however, such as mixed microbial cultures in which fast-famine periods are alternated, have proven to generate high PHA accumulation and productivity while avoiding the need for sterility during the process [87]. The intracellular location of the product determines the particular characteristics of its costly downstream processing due to the fact that cell lysis and dissolution of the polymer in non-ecofriendly organic solvents are needed for polymer purification. Much work has been done to increase the cost-effective production of PHAs; this includes metabolic engineering, bioprocess optimization, and the aforementioned design of mixed culture strategies, which considerably reduce operating costs [88,89].

PGA is currently produced by bacteria by means of submerged or solid-state fermentation (SSF) [90]. Different Mw PGA (from 100 to >1000 kDa) and enantiomeric compositions of the polymer can be produced via different species, carbon sources, and feeding strategies [35]. Productivity and purity may vary dramatically according to culture conditions such as ionic strength, aeration, temperature, and culture time. Production costs remain high, and this hinders the rollout of a wide range of PGA applications; consequently, recent research focuses on metabolic engineering aimed at precursor overproduction (glu) by means of inexpensive carbon sources [91]. Additionally, SSF, a less power-demanding culture strategy, has provided encouraging results; it has therefore been proposed as an economical alternative to PGA production [91,92]. PGA can be processed downstream by precipitation through reduction of solubility (i.e., ethanol addition), via precipitation by complex formation with cations, or by filtration [93].

### 3.2. Bacterial Biopolymer Production from Renewable Sources

**Figure 5:**
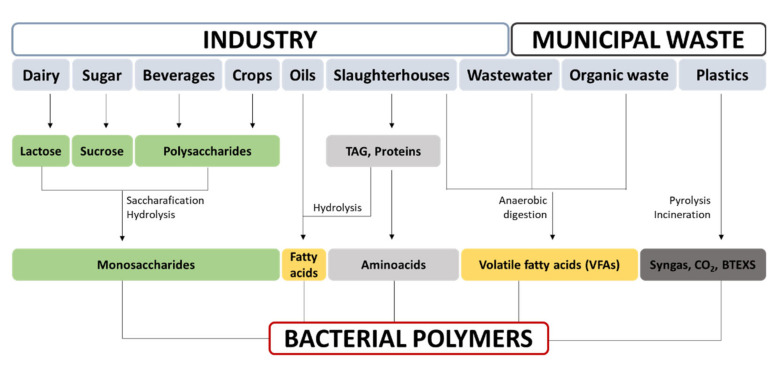
Flowchart of industrial and municipal upcycling of residues into bacterial polymers. Box colors indicate the highest percentage of carbon source in composition: green (saccharides), yellow (lipids, fatty acids), light grey (aminoacids), and dark grey (recalcitrant compounds: syngas, CO_2_, aromatics, and BTEXS. BTEXS includebenzene, ethylbenzen, toluene and xylene. Processes or treatments transforming the raw residues into bacteria assimilable substrates are indicated.

Sustainable production of bacterial biomaterials relies on the use of inexpensive, alternative, and renewable carbon sources as feedstock. Hence, byproducts from other processes and waste streams for use as substrates and the integration thereof into biorefineries would significantly enhance the economic competitiveness of bacterial polymers. For instance, industrial- or pilot-scale production of PHAs and BC is now a reality in waste management plants. These strategies are summarized in Figure 5.

Industrial food waste constitutes one of the principal feedstocks employed for biopolymer production. These sources are frequently acidic and of undefined composition, which pose environmental issues; furthermore, they have a negative impact on the environment when disposed of. Although they are suitable for microbial fermentation, their complex nature means that they are rarely used directly in culture media [88]. Previous treatment might therefore be required, depending on the composition, concentration, and purity of the waste as well as the microbial biocatalyst applied in the process [94]. Table 4 shows the suitability of wastes from different food industries for the production of bacterial biopolymers.

In this sense, whey is highly relevant. It is the main byproduct of dairy industries, and its estimated global annual production is over 100 × 10^6^ tons. Moreover, 47% of this waste is being poured straight down the drain [95]. In any case, the capacity to metabolize lactose is not common among bacteria. Indeed, very few PHA- or BC-producing organisms are able to produce polymers from it. Exceptions include some strains of *B. megaterium*, *Burkhodelia cepacea*, or *Methylobacterium* [96]. In order to identify new efficient PHA producers from whey, in silico prospection has proven to constitute a successful strategy. For instance, *Caulobacter segnis* DSM 29236 accumulates 37% of cell dry weight (CDW) in PHB, producing 9.3 g L^−1^ in fed-batch cultures [97]. Conversely, the lactose monomers, glucose and galactose, are commonly mineralized by many bacteria; consequently, a hydrolytic pretreatment could broaden the range of bacteria capable of fermenting such a waste stream. For example, BC production in diluted hydrolyzed whey media resulted in 0.25 g L^−1^ day^−1^ BC productivity by *K. xylinus* PTCC 1734 [85].

Entire or partial residues from crops are also predominant among biowastes produced worldwide [98]; they are frequently applied in sustainable processes for bacterial biopolymer production. A saccharification pretreatment is often needed to hydrolyze the complex carbohydrates present in such lignocellulosic residues. For instance, rice straw requires alkali treatment followed by enzymatic hydrolysis for PHA production, yielding 3.4 g L^−1^ of PHA in the case of *Burkholderia cepacia* JCM15050 [99]. Alternatively, this raw material can be pretreated with acids in combination with enzymatic hydrolysis, rendering xylose and glucose, used, for example, for PGA production by *B. subtilis* NX-2 [92]. Another example involves the hydrolysis of potato peels with nitric acid, the subsequent hydrolysate of which, fermented by *G. xylinum* ATCC 10245, yielded 0.65 g L^−1^ day^−1^ of BC [100]. BC can also be produced from an apple-pomace-based medium by *K. medenillensis* ID13488 fermentation (12.5 g L^−1^ of BC). This feedstock is a byproduct of the cider production industry, consisting of peels, seeds, cores, and pulp [62]. Recently, a pilot-scale study on BC production from saccharified oat hulls via sequential acid–alkali treatment exhibited outstanding yields of 80.5 tons of BC per 100 tons of hydrolysate when fermented by a microbial consortium [101]. SSF is often used for PGA production because it is more economical in terms of water consumption and energy power. For example, soybean meal and corn straw (CS) fermentation by *B. amyloliquefaciens* JX-6 was scaled up to 50 L, reaching 116 g PGA per Kg of residue [91].

Molasses, a well-known waste stream from the sugar industry, containing up to 50% sucrose, has frequently been investigated due to its potential for the production of biopolymers. Mixed and pure culture strategies have demonstrated the efficiency of this process in many bacterial strains such as *B. megaterium* BA-019, which has achieved 30.5 g L^−1^ [102]. Likewise, the high fermentable sugar content of molasses renders it a suitable feedstock for BC production. For instance, acidic pretreatment hydrolysis enhanced BC production of *G. xylinus* subsp. *sucrofermentans* up to 5.3 g L^−1^ in a corn step liquor (CSL)-molasses medium [103]. Finally, molasses directly fermented upon supplementation with monosodium glu waste liquor (MGWL) by *B. subtilis* NX-2 yielded a 50 g L^−1^ titer of PGA [104]. Additionally, nonsterile SSF of *B. subtilis* NX-2 immobilized onto sugarcane bagasse and used for cane molasses fermentation displayed an average titer of 90.6 g L^−1^ [105].

Interestingly, oil-derived feedstock such as household or industrial wastes can be used as a carbon source without the need for pretreatment to produce PHAs [106]. Some studies have demonstrated that *C. necator* H16 can use waste oil from sesame, palm, and sunflower to produce up to 105 g L^−1^ of PHB [107,108,109]. Furthermore, mcl-PHA production from used cooking oil has been tested [110]. Interestingly, supplementation of the standard BC production medium (HS) with 1% rapeseed oil raised BC production 6-fold, from 1 to 6 g L^−1^, in *K. xylinus* DSM 46602 cultures; this was due to certain physical phenomena, e.g., favoring oxygen exchange within deeper layers of cellulose [111].

## 4. Major Biomedical Applications of Model Bacterial Biopolymers

### 4.1. Drug Delivery Systems

Drug delivery systems constitute an area of biomedicine that is growing exponentially because these systems present numerous advantages over conventional formulations, such as increased drug solubility, bioavailability, and shelf life. They also reduce systemic side effects as systems can be engineered to target specific tissues or cells [145]. Polymeric materials are garnering much attention in this field because they provide delivery systems exhibiting stealth properties that help to evade early macrophage phagocytosis and immune response to carriers [146]. Furthermore, release kinetics can be tuned by varying the Mw of the polymer or through chemical modifications to meet specific needs (see Section 5).

Different formulations exist for polymeric drug delivery systems. The most common are particles, namely, capsules and spheres, either at the micro-scale or the nano-scale, due to their ease of preparation, their high drug loading capacity, and, in the case of nanoparticles, their ability to pass through different body barriers depending on the nature of polymer [147]. Particles have been developed to release a wide range of molecules, from small pharmaceuticals to therapeutic proteins. Although early research focused on synthetic polymers such as polyethyleneglycol or polylactic acid, degradable biopolymers have aroused interest in recent years due to their biocompatibility, biodegradability, and tailorable properties. Moreover, bacterial polymers may be more tailorable than synthetic polymers with regard to presenting properties such as degradation rates and, thus, release rates. In this sense, PHA *ter*-polymer P(3HB-*co*-3HV-*co*-3Hx) nanoparticles (NPs) loaded with immunosuppressant azathioprine (AZA) for systemic lupus erythematosus treatment displayed an enhanced in vivo therapeutic effect compared with PLA-loaded NPs or intravenous AZA administration. Due to their low degradation rate, a more sustained release over 15 days was achieved with PHA-AZA NPs compared with 4 days for total release for PLA-AZA NPs. Furthermore, PHA-AZA NPs showed a better biosafety profile due to a lower accumulation rate in the kidneys and less necrotic tissue in the liver [148]. In addition, the diversity of bacterial polymers can help to develop new routes of administration. As an example, PGA-functionalized chitosan nanoparticles (CS-PGA NPs) were used to develop oral formulations of insulin, in contrast with the standard transdermal delivery. Polymeric NPs were able to resist harsh gastric conditions, while the PGA coating increased intestinal uptake of the NPs via calcium-sensing receptors and amino acid transporters. Furthermore, in vivo assessment in Sprague–Dawley rats showed that CS-PGA NPs produced an increase in cumulative hypoglycemia 1.7 times greater than standard subcutaneous insulin administration, likely owing to a sustained release of insulin that was more similar to the physiological pattern [149].

BC is also thriving in the drug delivery field, particularly for cutaneous delivery systems, due to its high water-holding capacity (WHC). Interestingly, BC is naturally produced as a hydrogel with high water content and a high aspect ratio, which creates a suitable environment for the formation of BC-bioactive molecules. BC hydrogels are generally incubated with the targeted drug, which becomes trapped in the BC network, is retained via H-bonding with the hydroxyl groups, and can be subsequently released in the desired area [13]. For example, the antibiotic drug tetracycline hydrochloride was loaded onto BC membranes, which exhibited sustained release, prolonging the antibacterial effects of cutaneous administration of the drug [150]. Likewise, the anti-inflammatory diclofenac has been loaded into BC membranes; however, it displayed a very rapid release profile (>90% in 30 min) [151].

### 4.2. Tissue Engineering

Tissue engineering is the area of biomedicine dealing with the regeneration of tissues, which generally involves the use of scaffolds, porous matrices that mimic the native ECM and temporarily support cell and tissue growth until their degradation after implantation. Key factors affecting the success of scaffolds engineered for cell growth involve: (i) mechanical properties similar to those of the native tissue, as these properties are known to drive cell differentiation and adhesion [53]; (ii) porosity of the scaffold, which is linked to the ability of cells to infiltrate and migrate, and neovascularization; (iii) ECM-like information in the form of adhesion motives or growth factors; and (iv) biodegradability, which, in many cases, is desirable. Bacterial polymers are becoming commonplace in this field because the abovementioned factors can be tuned with relative ease via biotechnological or chemical procedures (see Section 5). Typical scaffold fabrication techniques such as electrospinning, salt-leaching, solvent casting, and polymer crosslinking to formulate hydrogels (such as native BC), as well as emerging 3D scaffold printing, are revised elsewhere [152,153].

Bacterial polymers are increasingly used for tissue regeneration. By way of an example, for bone regeneration, macroporous scaffolds made up of PHB/BC blends by means of salt leaching resulted in an interconnected porous structure with a pore size of 5–50 µm and a Young’s modulus of 1.2–14 GPa, within the range of bone rigidity (5–10 GPa). These structures supported 3T3-L1 preadipocytes, and BC enhanced in vivo differentiation into osteoblasts [154]. Other studies have demonstrated that PHB/nano-hydroxyapatite (PHB/nHA) composite scaffolds support and promote osteoblast cell growth [155]. In reference to skin regeneration, bacterial biopolymers were used to form porous BC scaffolds with gelatin-coated nanofibers, which displayed good adhesion and proliferation of keratinocytes, leading to 94% in vivo regeneration within 2 weeks in mice [156]. For cartilage regeneration, hydrogels generated by the crosslinking of thiolated PGA and glycyl-methacrylate-grafted PGA were formed. The resulting hydrogels showed a porous structure, elasticity (withstanding a 70% mechanical strain), and a compression modulus (up to 749 kPa) suitable for the recovery of cartilage [157]. For cardiac regeneration, P(3HO-co-3HD)/polycaprolactone (PCL) blends were designed to overcome the processability of mcl-PHA while enabling alteration of the mechanical properties of PCL. Additionally, 2D porous patches (100 μm pore size) were formulated by solvent casting, resulting in more effective mechanical properties (200% ε_b_, 0.02–0.05 MPa σ_t_, 1.5 MPa Young’s Modulus) than those of the targeted tissue, capable of resisting the long-term stress/strain the myocardium is subjected to. Moreover, on seeding the scaffold with murine cardiac progenitor cells, the implanted device in mice showed good cell attachment and in vivo proliferation [158].

### 4.3. Vascular Grafts, Cardiac Valves, and Vessel Stents

Vascular diseases frequently necessitate a bypass or a vascular graft in order to properly redirect blood flow. Although large artificial vascular grafts are routinely used in clinical practice, small diameter grafts still pose a challenge, and autologous implants remaining the gold standard. For artificial grafts, nanosized (<1 µm) structures are known to cause less coagulation or platelet adhesion than larger ones [159]. Therefore, BC has gained attention in this field due to its nanosized mesh structure. In a recent study by Wan et al., BC/cellulose acetate (CA) grafts with different diameters and polymer ratios were obtained at different fermentation times of *K. xylinus* upon a cylindrical CA electrospun structure. Grafts with a 6.1% (wt) BC content showed increased levels of tensile strength (1.2 MPa) and Young’s modulus (3.5 MPa), with values comparable to those of human umbilical veins. Results showed that BC/CA displayed better biocompatibility than BC or CA grafts separately as a lower inflammatory response and a thinner fibrotic capsule were observed [160].

Valve conditions frequently require surgical replacement with mechanical valves or homo- or xenografts. Although mechanical valves display better structural resistance, thrombotic complications constitute the main drawback. To address this issue, metallic valves have been coated with polymers to enhance biocompatibility. Due to their flexibility, mcl-PHAs are suitable polymers for valve coatings. For instance, when trileaflet-engineered valves of copolymer P(3HHx-*co*-3HO) were seeded with autologous ovine cells and implanted in the pulmonary position of lambs, no thrombus was formed, and only mild stenosis was observed for 120 days [161]. In another study, decellularized porcine aortic valves were coated with copolymer P(3HB-co-HHx) and implanted in the pulmonary position in sheep. The coated valves resulted in improved tensile strength and reduced calcification, promoting repopulation with the native host’s valve tissue, when compared with noncoated valves [162].

Other coronary diseases require implants such as cardiac stents, which have long been used to keep occluded vessels open; vascular congestion is a common condition derived from atherosclerosis. Although stents are fabricated from metals, polymer coatings may reduce the risk of thrombus. For instance, nitinol-PHA-coated stents displayed a lower inflammatory response as well as the absence of parietal thrombi and thinner intima thickness at the site of implantation when compared to nitinol implants; this points to PHA as a powerful coating material for vessel stents [163].

### 4.4. Wound Healing

An aging population, with the resulting increase in chronic wounds and ulcers, highlights wound treatment as another significant field in biomedicine. Wound healing is a complex dynamic biological process that implies ECM regeneration, cell growth, and secreted signaling factors. Wound dressings are now known to play an active role in healing as they substitute skin function during regeneration. Ideally, they should provide a moist environment, thermal insulation, and effective oxygen circulation; moreover, they should ensure liquid drainage and epithelial migration, aid in the absorption of wound exudates, and provide wound protection from bacterial loads [13].

BC meets most of these requirements or can be modified to this end (i.e., antibacterial activity; see Section 5). In particular, BC has demonstrated its effectiveness for burn patients because moisture is crucial for rapid healing. A recent prospective randomized clinical trial compared BC dressings (Epiprotect^®^ S2Medical AB, Sweden) with the standard silver sulphadiazine treatment of partial-thickness burns. The results suggested that BC dressings are the better first choice, as seen in rapid healing rates, low pain scores, and fewer dressings used [164]. Furthermore, wound healing is the field in which BC has largely reached the commercial level; several BC-based dressings are currently sold on the market. For instance, Superabsorb^®^ X+ PHMB is the first BC combined drug delivery–wound healing patch on the market; it possesses healing and antimicrobial properties as it releases antiseptic polyhexamethylene biguanide. Other BC-based wound dressings are marketed as temporary skin substitutes (Biofill, Membracell, Bionext^®^) or ulcerous wound dressings (xCell, Bionext^®^, Nanoskin^®^); these have recently been reviewed elsewhere [165].

Other biopolymers are also used for wound treatment. Dressings constructed from copolymer P(3HB-*co*-4HB) films and electrospun nanofiber membranes cultured with allogenic fibroblasts were assessed in a skin defect model; cells were observed to secrete ECM, forming a layer that promoted migration of the cells in the vicinity of the wound and thus, wound closure. This resulted in healing times 1.4 times faster than for noncarrying allogenic cells and 3.5 times faster than the ones healed under scars [166]. PGA has also been studied for wound dressings, although in combination with other polymers. Hydrogels of PGA with other polymers, for instance, gelatin, have been studied for wound regeneration. The PGA-gelatin hydrogels properly absorbed exudates and showed accelerated wound repair, outperforming the untreated group [167].

### 4.5. Sutures and Biological Glues

Sutures are designed to hold tissues together and accelerate healing processes while minimizing scar formation. There is currently a growing demand for suture materials as surgical procedures are increasing in number and because material requirements differ for each procedure [168]. Work is underway to develop second-generation sutures displaying bioactive functionalities, i.e., antimicrobial sutures for preventing surgery-associated infections [169] or absorbable sutures. Microbial polymers have found a niche in this field due to their tensile properties, which help to resist tissue-induced stress, and because of their ease of functionalization. For instance, P(4HB) sutures were the first FDA-approved PHA-based device; they exhibit a tensile strength that outperforms that of commercially available monofilament sutures (545 MPa vs. 410–460 MPa for polypropylene sutures), and they are highly flexible (1000% ε_b_) [69]. These sutures are now marketed under the ThephaFLEX trademark.

Alternatively, biological glues connect tissues without the need for sewing. PGA-gelatin coupled with 1-(3-dimethylaminopropyl)-3-(ethylcarbodiimide) hydrochloride (EDC) displayed better implant–tissue interface adhesion than fibrin-based glues in a rat model. The mixed glues showed a greater bonding strength and shorter gelation times as the molecular weight of gamma-PGA or gelatin increased [170].

## 5. Increasing Bacterial Polymers’ Structural Diversity by In Vivo and In Vitro Functionalization

The high degree of applicability of bacterial biomaterials can be enhanced by tailoring their properties; this can involve improving their mechanical properties or modulating their thermal properties for easier manipulation of the materials. Furthermore, new functionalities that do not exist in the native polymer can be conferred. For instance, degradation rates can be modulated by changing the polymer structure, which can lead to different release profiles; polymeric matrices can be enhanced for cell attachment, resulting in a faster migration rate for tissue engineering, or antimicrobial activity can be conferred for infection treatment (i.e., wound healing) or prevention (i.e., implants).

**Figure 6:**
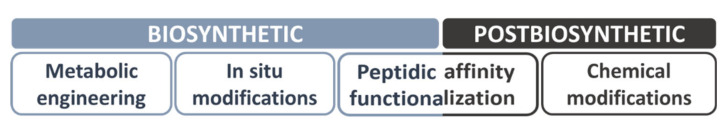
Flowchart of the different approaches used in the literature to modify bacterial polymers.

The following subsections show the strategies (Figure 6) for modifying the properties of biopolymers, mainly BC and PHA; in this sense, more detailed information is provided in the literature.

### 5.1. In Vivo Functionalization Approaches

This section addresses two different approaches: one based on the use of genetic engineering and synthetic biology tools to modify the biosynthetic machinery, thus varying the resulting polymer (metabolic engineering); the second approach is based on the inherent ability of bacteria to incorporate exogenously provided molecules into the polymer (in situ modifications).

#### 5.1.1. Metabolic Engineering

The potential to edit and redirect the cell system using metabolic and genetic engineering tools combined with bioprocess engineering strategies has led to the creation of rationally designed PHAs for targeted applications [29,31]. It is now possible to obtain PHA homopolymers, random and block copolymers, or functional and graft PHA polymers by controlling the carbon flow by manipulating the bacterial PHA synthesis metabolism [171,172]. These strategies have resulted in polymers with enhanced thermal and mechanical properties as well as those presenting new functionalities. For instance, homopolymers obtained through weakened β-oxidation mutants show improvements in tensile strength, elongation at break, and Young’s modulus [67]. Mcl-PHA copolymers showing a higher percentage of long-chain length monomers, such as C12 and C14, exhibit significantly improved thermal and mechanical properties [67]. These polymers showed higher crystallinity, but they behave as thermoplastic elastomers, displaying good tensile strength and desirable elongation at break. They display an increased Young’s modulus, a fact that has been shown to improve the processing and molding of the polymer [67,173]. P(3HB-co-3HHx) is one of the most promising copolymers as it has shown increased flexibility and high biocompatibility properties, thus rendering it appropriate for biomedical applications such as tissue engineering [174]. In addition, the P(3HB-co-4HB) copolymer constitutes a very interesting polymer since its crystallinity and degradability properties can be modified by adjusting the 4HB content [175]. This monomer has been reported to increase the elongation to break by up to 1000%, which increases its applicability in medical fields [69].

Furthermore, the ability of some bacteria to incorporate R-HA-CoA, bearing functional groups from related substrates, gives rise to great monomeric structural diversification by both biosynthetic and postbiosynthetic chemical modifications [31]. Related carbon sources have been used to obtain PHAs containing double or triple bonds [176], monohalogenated [79], aromatic [73,74], thioether [177], thioester [77], cyano, or nitro [76] side groups, among others.

In *P. putida*, the combination of the metabolic engineering strategies and the use of a cofeeding strategy with 6-acetylthiohexanoic acid resulted in a new polymer known as PHACOS, which possesses controllable thioester content [77]. The thioester group confers on the PHACOS polymer a novel antimicrobial functionality; it displays both in vitro and in vivo bactericidal activity against methicillin-resistant *S. aureus* and inhibits biofilm formation [178]. In light of these results, metabolic engineering clearly constitutes a powerful tool for the development of new intrinsically bactericidal polymers.

Metabolic engineering in BC-producing strains remains to be further explored. This is likely due to the lack of fundamental knowledge of these bacteria, as they were sequenced only a few years ago (2010) [23], and to the fact that the genetic toolkits required to enable their modification via synthetic biology approaches have only recently been developed [179,180]. Transformation with a unique gene, curdlan synthase, responsible for the synthesis of curdlan, a water-insoluble linear beta-1,3-glucan from *Agrobacterium* sp. ATCC 31749, into *G. xylinus* AY201 sufficed for in vivo synthesis of a composite of BC-curdlan, with modified morphology and porosity. As curdlan covered the BC pores, the resulting composite displayed reduced water permeability and greater hydrophobicity than BC [181]. Considering the ability of cellulose synthases (BcsAB) to recognize UDP-Glc-related substrates, another possibility involves the combination of feeding and genetic engineering strategies. For instance, a fluorescent BC was synthetized by feeding *K. sucrofermentans* with 6-carboxyfluorescein-modified glucose [182]. Additionally, due to the fact that BcsAB is capable of recognizing UDP-N-acetylglucosamine (NAcGlc), a transcription unit comprising 3 genes from *Candida albicans,* enabling UDP-NAcGlc synthesis, was transferred to *G. xylinus* 10245. The resulting strain was able to synthesize a polymer with up to 20% NAcGlc content when grown in NAcGlc-containing media [44]. Subsequently, the system could be tuned in terms of production by swapping promoters [180]. Furthermore, the resulting polymer was susceptible to lysozyme degradation and, thus, displayed novel biodegradable characteristics in vivo [44] and supported the attachment, proliferation, and differentiation of hMSCs into cartilaginous tissue [183].

These preliminary studies are paving the way towards rational approaches aimed at increasing the mechanical and functional properties of BC; metabolic engineering of BC-producing bacteria can, therefore, be expected to become much more commonplace in the coming years.

PGA properties have also been modified via metabolic engineering, namely, Mw and enantiomeric composition. Different Mw polymers were achieved through the expression of Pgs synthetases from different origins in *B. subtilis* as a host. For instance, low Mw (29–34 kDa) was produced with *B. anthracis* pgs, medium Mw (170–660 kDa) with pgs from *B. amyloliquefaciens*, and high Mw (up to 8 500 kDa) with its native operon [184]. This is noteworthy because different Mw PGAs would result in different applications. For example, high Mw is used as an immune-stimulating agent [185], while low Mw PGA is preferred for drug delivery formulations [186]. Furthermore, the combination of the different pgs synthetases with glu racemases provided different stereochemical compositions ranging from 3% to 60% D-glu [184]. Another approach was used to tailor the enantiomeric composition of PGA produced in heterologous host *C. glutamicum* F343 by introducing glu racemase from *B. subtilis* under different expression strength promoters, varying the L-glu content from 36.9% to 97.1% [40].

#### 5.1.2. In Situ Modifications

A simple, cost-effective method of bacterial biopolymer modification involves the in situ supplementation of the medium with additive chemicals in the culture medium, which can be incorporated directly into the growing polymer chains. This approach is widely reported in the literature for BC modification, for example, and has been extensively reviewed elsewhere [187]. Indeed, this approach can be harnessed for incorporating bioactive molecules (i.e., antimicrobials) into BC in an economical manner, although there is a need to address certain concerns regarding the cytotoxicity for the producing bacteria, the effects on the already low yields of BC production, as well as the compatibility of the incorporating molecules with the restrictive fermentation conditions of the *Komagaetibacter* genus. For these reasons, very few of the many antimicrobial molecules have been incorporated by means of this approach.

Butchosa et al. first reported the bactericidal activity of BC on the incorporation of partially deacetylated chitin nanocrystals (D-ChNCs). D-ChNCs were formed by chitin treatment with NaOH and subsequently added to an *Acetobacter aceti* AJ-12368 culture medium. The resulting BC pellicles had only an 8% content of D-ChNCs due to the toxicity exerted towards the bacterial producer [188]. To overcome the bactericidal effects of CS on BC-producing bacteria, a rotating dynamic culture strategy was employed to produce in situ fabric-embedded CS/BC hydrogel sheets in a horizontal rotating bioreactor, resulting in composites with bacteriostatic activity against *Staphylococcus aureus* and *E. coli* [189].

Bioactive glass nanoparticles (NBG) have also been studied as additives to BC-producing media for *G. xylinus* ATCC 10245. Bioactive glass based on SiO_2_, CaO, and P_2_O_5_ was incorporated homogenously into growing BC chains. The resulting composite, BC–NBG, presented an enhanced antibacterial activity when compared to NBG, displaying bacteriostatic activity at concentrations as low as 50 mg mL^−1^ for the Gram-negative pathogens *E. coli*, *P. aeruginosa*, *Proteus vulgaris*, and *Kleibsella pneumonia* and the Gram-positive bacteria *B. subtilis* and *S. aureus*. Moreover, the NBG additive also enhanced BC productivity by 2.3 times as a result of its buffering capacity, which counteracted inhibition by gluconic acid conversion from glucose [190].

An economical procedure was performed by Chen et al., who took advantage of the naturally occurring antibacterial molecules present in plants. By supplementing BC-producing medium with mulberry leaf hydrolysate, they achieved incorporation of its flavonoids, mainly rutin and quercetin, into BC pellicles. Although the BC synthesis inhibitors present in the extract reduced the BC yield, the resulting pellicle presented bacteriostatic activity against both Gram-negative (*E. coli*) and Gram-positive bacteria (*S. aureus*) [191].

Conversely, intracellular polymers (i.e., PHAs) are more difficult to chemically functionalize in situ during production. One example consists of PHB–AgNPs composites formed in vivo. Upon addition of silver nitrate in cultures of *C. necator* grown for 24 h, this bacterium showed an inherent capacity to reduce it to AgNPs. Furthermore, the resulting AgNPs were dispersed among the PHB granules, which upon extraction and formulation into films, displayed antibacterial activity against *Listeria monocytogenes* and *Salmonella enterica* [192].

### 5.2. In Vitro Functionalization Approaches

This section addresses two different approaches: one based on the ability to functionalize biopolymers with peptides using tags derived from their microbial metabolic machinery (peptide affinity-based functionalization); a second approach employs polymer chemistry to diversify the chemical structure of the material driven by the target application (chemical modifications).

#### 5.2.1. Peptide Affinity-Based Functionalization

PHAs have been proposed as a material that can easily be functionalized by tagging granule-associated proteins (GAPs). The strong binding of GAPs both to in vivo and in vitro granule beads, whether natural or artificial, and the fact that they can be cost-effectively produced from various microorganisms by large-scale fermentation have all boosted the emergence of many biotechnological and medical applications. By fusing the gene coding for a protein of interest to any GAP, the PHA beads can be functionalized to display new tailor-made properties [178]. Phasins, PHA synthases, and depolymerases (or their PHA-binding domains) have been used as affinity tags for the display of heterologous proteins on PHA both in vivo and in vitro. The advantage of the in vivo procedure comes from the fact that both the PHA biosynthesis and the protein expression and immobilization take place simultaneously, reducing time and costs. Moreover, the in vivo PHA biosynthesis provides the cytoplasm a more oxidizing environment that favors native-like disulfide bond formation, and, in fact, the recombinant human tissue plasminogen activator, containing nine disulfide bonds, can be properly folded purified from the supernatant fraction after the in vivo immobilization on the granule, making use of PhaP1_Reu_ as an affinity tag and a thrombin cleavage site as a linker [193]. However, an in vitro approach in which both fusion proteins and polymers are separately purified and subsequently bound ensures purity standards and tight control of the protein load, which is more suitable to biomedical applications. In any case, these approaches, widely reported for PHA and, to a lesser extent, for BC, still remain largely unexplored for PGA functionalization.

Functionalized PHAs are increasingly being used as drug-delivery systems. The human α1-acid glycoprotein [194], the epidermal growth factor receptor-targeting peptide [195], the RGD4C peptide [196] tagged to PHA nanoparticles by means of the phasin PhaP from *Aeromonas hyrophila*, or the synthase PhaC from *Ralstonia eutropha* have all been tested as tumor-targeting carriers, whereas the mannose receptor of macrophages immobilized to the PHA nanobeads by PhaP has been proven to specifically bind macrophages [194].

PHA granules with a tag-based oriented display of antigen or antibodies have been successfully generated and can be employed for research or clinical diagnostic purposes. The PHA-binding domain of PhaZ depolymerase from *Alcaligenes faecalis* (SBD_Afa_) was fused to a severe acute respiratory syndrome coronavirus envelope protein and used in subsequent immunoassays for diagnosis [197]. Similarly, the same binding domain was fused to streptavidin and used to immobilize pathogen–biotin-labeled DNA probes on PHB beads that were formulated into a microarray capable of detecting the DNA of specific pathogens even in the presence of other microorganisms [198]. In another study, mouse interleukin-2 (IL2) and the myelin oligodendrocyte glycoprotein (MOG), fused to PhaP and attached to the polyester beads, were employed for the specific and sensitive detection of antigen-specific serum antibodies by fluorescence-activated cell-sorting (FACS) technology [199]. On the other hand, the immune-dominant bovine tuberculosis antigens ESAT6, CFP10, Rv3615c, and Rv3020c were translationally fused to the PHA synthase from *P. aeruginosa*, succeeding in the formation of antigen-displaying polyester beads that demonstrated to be a cost-effective tool for skin testing detection of bovine tuberculosis [200].

For tissue regeneration applications, PHA’s lack of recognition sites for cell attachment needs to be compensated by immobilizing biomolecules, such as growth factors, therapeutic proteins, ECM proteins, or the cell-binding domains of ECM. Tailoring the properties of the material surface enables improved biocompatibility and controlled cell adhesion. For this purpose, the phasin PhaP from *Aeromonas hydrophila* has been genetically fused to ubiquitous Arg–Gly–Asp (RGD) or the laminin-derived Ile–Lys–Val–Ala–Val (IKVAV) motifs, purified and bound to artificial particles in vitro. These fusion proteins are capable of being recognized by the transmembrane integrins, which are then activated, inducing and promoting cell adhesion/proliferation. This strategy has been developed for implant tissue engineering in mouse fibroblasts [201], human bone marrow mesenchymal stem cells [202], and neural stem cells [203].

Finally, affinity-tagged PHA beads have been employed as vaccine delivery systems based on antigens displayed on bioengineered bacterial polyesters, which have been proven to stimulate cellular immune responses both for bacterial [13,204] or viral [205,206] infections. Moreover, they have been applied as antibacterial surfaces using the PhaP tag to effectively coat the antimicrobial peptide tachyplesin I (Tac) on a PHBHV polymer as an in vivo skin wound model, accelerating wound healing by efficiently inhibiting the growth of both Gram-negative and Gram-positive bacteria [207].

In the case of BC, a broad family of cellulose-binding domains (CBDs) exists naturally as part of fungal and plant cellulases as well as in scaffolding units of bacterial cellulosomes. They vary in their capacity to bind to crystalline BC, amorphous BC, or both types of polymer. They also vary in the reversibility of the bonding as well as in their specificity, with many tags being able to bind to other types of polysaccharides such as chitin. A detailed revision of CBDs is provided elsewhere [208] or in the carbohydrate-binding domain database.

The use of CBDs has long been widely reported for tagging [209], immobilization of proteins of industrial interest [210], and fiber modification [211], but not to the same extent for the incorporation of biomedical molecules. By fusing the gene coding of a protein of interest to any CBD, cellulosic materials can be functionalized to display bioactive activities.

For tissue engineering applications, BC’s 3D network structure provides the environment to allow cell growth within the matrix. However, cells attach weakly onto bare BC. This can be improved by using adhesion molecules. In this sense, the CBD from the cellulosomal scaffolding protein A of the bacteria *Clostridium thermocellum* (CBD_ther_) has been fused to RGD peptides, purified and immobilized into BC matrices. These fusions have successfully enhanced adhesion/biocompatibility in BC with mouse fibroblast embryo culture [212] and human microvascular endothelial cell proliferation [213], with demonstrated hemocompatibility of the resulting material [214]. Likewise, CBD_ther_ was fused to IKVAV peptides, resulting in better adhesion to functionalized BC of both neuronal and mesenchymal cells [215].

Moreover, a relatively unexplored domain involves the antimicrobial coating of BC via CBD functionalization. A T4 phage lysozyme was fused to the CBD of a scaffolding protein from the cellulosome of *Cellulomonas fimi*. The resulting antimicrobial fusion protein was attached to commercial cellulose gauzes, which then displayed bactericidal activity against both Gram-positive and Gram-negative bacteria [216]. A shortened CBD motive of only 7 amino acids was fused to a short antimicrobial peptide (12 amino acids), resulting in membranes that displayed up to 4 logarithmic units of reduction against Gram-positive *S. aureus* and 1 logarithmic unit reduction against Gram-negative *P. aeruginosa* [217].

#### 5.2.2. Chemical Modifications

There are many possibilities for chemical functionalization to increase the diversity of bacterial polymers possessing properties tailored to the target applications. Strategies can be based on blending, grafting/crosslinking, and curing. These techniques are generally combined to obtain the final materials, with properties for specific applications [13,204,218,219,220].

Blending strategies result in materials formed via physical interactions by means of mixing different polymers. The process of blending usually involves solvent-casting and melt-compounding methods [221]. In the case of BC, a major drawback is its poor solubility in both polar and nonpolar solvents. Previous modifications of BC, such as acetylation or grafting with polar molecules, can enhance its solubility. Thus, very few examples of BC blending can be found in the literature.

Grafting is a method for covalently binding molecules (or other polymers) to a polymer chain to produce modified (or newly conferred) physicochemical or functional properties in the resulting material. Different methods have been developed to obtain graft copolymers derived from bacterial biopolymers; some of these involve chemical, photochemical, radiation, plasma-induced, and enzymatic grafting techniques. Surface properties can also be modified with the use of chemical reactions. In this sense, the abundance of hydroxyl groups on the surface of BC makes them susceptible to modification, transforming them into carboxylic acid, amine, aldehyde, or thiol groups. Further modification of these groups could lead to the grafting of a wide range of molecules such as proteins, polymers, metal nanoparticles, and antibiotics [222]. Plasma treatment is a grafting technique consisting of the dispersal of an ionized gas; the procedure introduces functional groups onto the surfaces due to the high reactivity of the ions and electrons. Interestingly, it only modifies the surface layer without affecting the bulk properties of the polymer [204].

Also highly relevant is chemical crosslinking, which consists of the covalent bonding of monomers onto a polymer chain in the presence of crosslinkers, forming tridimensional networks [223]. This strategy has been explored to obtain a polymeric matrix with enhanced properties aimed at conferring to it antimicrobial activity by adding another component in a further step.

Curing is a technique in which an oligomer polymerizes to form a coating that adheres to the substrate by physical forces. The most popular curing method for polymer composites is thermal curing. It is widely used for preparing biocomposites due to its application in industry, including the automotive, construction, and furniture sectors. These biopolymers are reinforced with other compounds such as resins [224] or zirconia [225] in order to provide better mechanical properties. However, they are not commonly used to obtain materials for biomedical applications.

Finally, the preparation of composites based on bacterial biopolymers involves techniques such as polymer blending, crosslinking polymer matrices, and branched copolymers, along with the incorporation of additives such as plasticizers, reinforcement by inorganic fillers, binary salt systems, and impregnation with ionic liquid doping of nanomaterials [226]. These functionalized composites can incorporate antibiotics, organic or inorganic compounds, antimicrobial peptides, or cationic agents to obtain antimicrobial activity [13,204]. Table 5 provides revised information on chemical modifications of bacterial biopolymers.

## 6. Regulatory Aspects and Transferability into Clinics

The field of nanomedicine faces several challenges for regulatory approval as a result of the unpredictability of nanobiomaterials with biological systems, which has been assessed so far in a case-by-case manner. The current lack of guidelines for nanomedicine research has recently been filled by the new Safe by Design (SbD) approach developed within EU nanomedicine projects. It is worth noting that the development of polymeric nanomaterials designed for nanobiomedicine should include a Safety by Design (SbD) evaluation, which is based on the identification and reduction of the risks regarding human health and environmental safety. A proposal of normative and regulatory requirements as well as the methodological approaches of SbD for nanobiomaterial development are described in the framework of the GoNanoBioMaT project from the Swiss Federal Laboratories for Materials Science and Technology (EMPA) [273,274].

However, it is remarkable that very few products have reached the commercial level despite the years of research and the ever-growing number of patents regarding bacterial polymers for biomedical applications.

For instance, the first company to obtain FDA approval for PHA-based devices was the Thepha Inc company (USA). It obtained its approval for P(4HB)-based sutures in 2004. Since then, they have developed other approved devices such as surgical films and composite meshes. Other commercial medical products based on PHA include the ElastoPHB biopolymer membrane system for repairing soft and cartilage tissue defects (BIOMIR Service JSC, Moscow, Russia).

Likewise, despite the ideal properties of BC in the field of wound healing and the generally recognized as safe (GRAS) status of BC producer strains, only a few commercial dressings have been approved by regulatory agencies and commercialized. Their main indications are as skin substitutes for burn and ulcer patients. Some examples are Biofill^®^ and BioProcess^®^, with antibacterial properties (Biofill, Brazil); Xcell^®^ and Xcell antimicrobial from Xylos (Xylos Corporation, PA, USA), which obtained the FDA approval in 2003; antimicrobial Ag-based Nanoderm^®^ (Axcelon Biopolymers Corp, Canada); Superabsrob X + PHMB, a drug delivery system of antiseptic PHMB (Activa Healthcare, L&R Company, UK); EpiProtect^®^ (S2Medical AB, Sweden); Membracell (Vuelo Pharma, Brazil); and Nanoskin (Innovatec, Brazil). Other applications with commercial use include dental implants (Gengiflex^®^ by Biofill).

In the case of PGA, the current market situation is focused on PGA production as an additive for personal care and cosmetic uses (i.e., SpecKare PGA by Spec Chem Industry, Nanjing, China; PGA by XI’AN Neo Biotech Ltd., Nanjing, China). For instance, in 2019, Cult Beauty (UK) launched a serum with PGA as the main active compound as a moisturizing agent. This lack of biomedical-based PGA products in the market, to the best of our knowledge, reflects the late development of PGA research regarding the other two polymers, as has been highlighted throughout this review.

## 7. Conclusions

This review provides insight toward the recent advances in bacterial biopolymer production by upcycling bioprocesses, their main uses in the biomedical field as nanomaterials, and, finally, the different approaches to structurally diversify these polymers to further expand their applications. Despite great advances in the design of cell factories and bioprocesses to enhance biopolymer production, challenges remain to gain economic competitiveness. Upcycling of industrial and municipal wastes is indeed a necessary approach to increase economic profitability while reducing the global environmental impact. The interest in bacterial polymers has grown exponentially in biomedical research, mainly in three domains: drug nanocarriers, implantable material coatings, and tissue-regeneration scaffolds or membranes due to their biocompatibility, biodegradability, and mechanical properties. Bacteria are able to produce a considerable diversity of polymer structures with a plethora of backbones, such as polysaccharides (e.g., BC), polyesters (e.g., PHA), and polyamides (e.g., PGA), the chemical structures and mechanical properties of which depend on the metabolic background of the cell biocatalyst and the feedstock and fermentation conditions. Here, we provide examples of BC, PHA, and PGA applications as nanocarriers for drug delivery systems and in tissue engineering, wound healing, vascular grafts, cardiac valves and vessel stents, sutures, and biological glues. Moreover, the natural diversity of bacterial polymers can be further broadened by means of metabolic engineering, in situ modifications, peptide functionalization, and chemical modifications, thus enhancing their properties or conferring new functionalities. Synergistic and multidisciplinary strategies based on cutting-edge technologies, such as synthetic and systems biology, combined with advanced materials technology, including blending, grafting/crosslinking, and curing, provide pathways for enhancing the structural and functional complexity of these biopolymers, thereby expanding the catalog of available biomaterials beyond that which exists in nature, as well as extending their potential applications as nanomaterials in the biomedical sector.

## Figures and Tables

**Table 1:** Main characteristics of the model bacterial polymers BC, PHA, and PGA.

	BC	PHA	PGA
Chemical structure	Polysaccharide (Figure 1A) Glucose (glc) homopolymer. Properties of the polymer depend on culture conditions Hydrophilic	Polyester (Figure 1B) High diversity. Polymer properties rely on its monomer combination Hydrophobic	Polyamide (Figure 1C) Anionic. D- or L-glutamic acid (glu) homopolymers, or D-/L-glu copolymers Hydrophilic
Industrial production prototype bacteria	Species belonging to *Komagataeibacter* genus, *K. xylinus*	High diversity Scl-PHA *Cupriavidus necator*, *Halomonas* spp. Mcl-PHA *Pseudomonas* spp.	*Bacillus* spp., *B. subtilis*
Precursors at industrial production level	Direct: sugars, preference depends on the species	Direct: fatty acids	Direct: glutamic acid
	Indirect: ethanol, converted into acetate, and, finally, glc through tricarbolxylic acid cycle (TCA) and gluconeogenesis (GNG) (Figure 2)	Indirect: sugars through TCA and de novo synthesis of fatty acids (Figure 3)	Indirect: sugars, through TCA and alpha-ketoglutarate (α–KG) conversion into glutamic acid (Figure 4)
Culture conditions for pure cultures industrial production	Submerged fermentation Mainly in static conditions for biomedical applications	Submerged fermentation Batch and Fed-Batch strategies	Submerged and solid-state fermentation
Downstream processing	Extracellular polymer. Easy, cheap purification, isolation, and alkali treatment	Intracellular polymers. Costly purification, cell lysis, release, and polymer isolation	Extracellular polymer. Precipitation by chelation, solubility reduction or filtration

**Table 2:** Mechanical and material properties of BC produced by different *Komagataeibacter* strains.

Producing Species	WHC ^1^ (%)	*E*^2^ (MPa)	σ_t_^3^ (MPa)	ε_b_^4^ (%)	CI^XRD 5^	Ref.
*K. xylinus*						
B12068	NR	3.73	NR	12.5	0.65	[58]
ATCC 10245	5400	2.87	0.36	18.6	NR	[59]
NBRC 13693	16,500	3.1	0.62	18.7	NR	[59]
ATCC 53524	NR	9.09	1.68	26.9	NR	[60]
ATCC 23760	18,000	1.5	46.9	2.5	0.85	[61]
*K. medellinensis*						
ID13488	7218	6.75	50	NR	0.89	[62]
*K. sucrofermentans*						
ATCC 700178	52,600	1.1	0.15	20.72	NR	[59]
DSM 15973	260	NR	NR	NR	0.87	[63]
*K. hansenii*						
ATCC 23769	45,000	1.26	0.12	17.9	NR	[59]
GA2016	700	NR	NR	NR	0.87	[64]
*K. rhaeticus*						
AF-1	14,000	3.2	46.9	1.5	0.89	[61]

Reported properties of hydrated BC produced in Hestrin–Schramm (HS) culture medium with glucose as the carbon source. ^1^ WHC:**** water-holding capacity. ^2^ *E*: elastic modulus. ^3^ σ_t_: tensile strength. **^4^** ε**_b_**: elongation at break. ^5^ CI^XRD^: crystallinity index. NR: not reported.

**Table 3:** Thermal and mechanical properties of some representative polymers from the PHA family.

Polymer	Copolymer Content (%)	*E*^1^ (MPa)	σ_t_^2^ (MPa)	ε_b_^3^ (%)	*T_m_*^4^ (° C)	*T_g_*^5^ (° C)	Ref.
PHA *(R)-alkanoic*							
P(3HB)	100	3500–4000	40	3–8	173–180	5–9	[68]
P(4HB)	100	70	50	1000	60	−51	[69]
P(3HB-*co*-3HV)	(97:3) (91:9) (75:25)	2900 1900 700	38 37 30	- - -	170 162 137	- - -	[70]
P(3HB-*co*-4HB)	(97:3)	NR ^6^	28	45	166		[71]
	(90:10)	NR	24	252	159		
P(3HB-*co*-3HHx)	(88:12)	1286	18.3	3.6	170		[72]
	(45:55)	1207	21.6	4.1	167		
P(3HD-*co*-3HDD)	(15.7:84.3)	103.13	5.24	88.30	77.62	32.49	[67]
*(R)-Aromatic*							
P(3HPhHHx)	100	NR	NR	NR	NR	−1.3	[73]
P(3HB-*co*-3H3PhP)	(89.5:8.9)	NR	NR	NR	135, 149	14.6	[74]
P(3HDD-*co*-3H5PhV)	(97.1:2.9)	93.9	2	37.38	81	−33.4	[75]
	(68.1:31.9)	48.7	3.15	32.2	75.84	−35.2	[75]
*(R)-Nitrogen*							
P(3H-p-nitroPV-*co*-3HN)	(4–7% N)	NR	NR	NR	56.4	−35.9, 28.7	[76]
*(R)-Sulfur*							
PHACOS	(16.5 to 77% thiolated side chains)	NR	NR	NR	--	−5	[77]
*(R)-Halogenated*							
P(3HB)-Cl	(22% Cl)	NR	NR	NR	134	2	[78]
P(FHB-*co*-HB)-F	(7% F)	NR	NR	NR	160.5 173.6	−0.8	[79]

^1^*E*: elastic modulus. ^2^ σ_t_: tensile strength. ^3^ ε_b_: elongation at break. ^4^ *T_m_*: melting temperature. ^5^ *T_g_*: glass transition temperature. ^6^ NR: not reported. P(3HB-*co*-3H3PhP): poly(3HB-*co*-3-hydroxy-3-phenylpropionate); P(3HPhHHx): poly(3-hydroxyphenylhexanoate); P(3HDD-3H5PhV): poly(3-hydroxydodecanoate-*co*-3-hydroxy-5-phenylvalerate; P(3H-p-nitroPV-co-3HN): poly[3-hydroxy-5-(4-tolyl)valerate]-*co*-3-hydroxynonanate; PHACOS: poly(3-hydroxyoctanoate-*co*-3-hydroxyhexanoate-*co*-6-acetylthioalcanoate).

**Table 4:** Sustainable production of bacterial polymers from industrial and municipal waste.

Waste Origin	Strain	Productivity (g L^−1^ day^−1^)	Type of Polymer	Ref.
Cheese whey				
	*Alcaligenes latus*	2.64	P(3HB)	[112]
	*Caulobacter segnis*	4.56	P(3HB)	[97]
	*Haloferax mediterranei*	4.04	P(3HB)	[113]
	*C. necator* DSM 545 (recombinant strain)	0.82	P(3HB)	[114]
	*K. sucrofermentans* DSM No 15973	2.7	BC	[115]
	*Acetobacter* strain ITDI 2.1 (recombinant strain)	0.1	BC	[116]
Cane molasses				
	*Pseudomonas*	2.17	(P3HO-*co*-3-HHx)	[117]
	*B. megaterium BA-019*	30.48	P(3HB)	[102]
	Mixed culture	10.93	mcl-PHA	[118]
	*A. xylinum* BPR 2001	1.77	BC	[103]
	*A. pasteurianus* RSV-4 yielded	0.51	BC	[119]
	*B. subtilis* NX-2	12.96	PGA	[104]
	*B. subtilis* NX-2	25.92	PGA	[105]
Crops				
Vinasse	*H. mediterranei*	5.04	P(3HB-*co*-3HV)	[120]
	*H. marismortui*	0.48	P(3HB)	[120]
Sugarcane bagasse	*C. necator*	3.16	P(3HB)	[121]
	*B. cepacia* IPT 048	11.28	P(3HB)	[122]
Grape pomace	*P. putida* KT2440	1.2	P(3HO-*co*-3-HHx)	[123]
Waste beer yeast	*G. hanseii* CGMCC 3917	0.514	BC	[124]
Apple pomace	*K. medenillensis* ID13488	0.177	BC	[62]
Potato peel	*G. xylinum* ATCC 10245	0.65	BC	[100]
Citrus peel	*K. xilynus* CICC No 10529	0.712	BC	[125]
Orange juice	*A. xylinum* NBRC 13693	0.421	BC	[126]
Litchi extract	*G. xylinus* CH001	0.18	BC	[127]
Citrus waste	*K. sucrofermentans* DSM 15973	0.515	BC	[128]
Coffee cherry husk	*G. hanseii* UAC 09	0.547	BC	[129]
Olive oil mills	*G. sacchari*	0.212	BC	[130]
Tomato juice	*A. pasteurianus* RSV-4	0.68	BC	[119]
Rice straw	*B. cepcecea USM* (JMC 15050)	1.95	P(3HB)	[99]
	*B. subtilis* NX-2	0.87	PGA	[92]
Soybean meal and Corn straw	*B. amiloliquefaciens* JX-6	116 (g kg^−1^)	PGA	[91]
Soybean straw	*B. amiloliquefaciens* NX-2S	65.79 (g kg^−1^)	PGA	[131]
Household and industrial oils				
Sesame	*C. necator H16*	31.32	P(3HB)	[107,109]
Sunflower	*C. necator H16*	35.04	P(3HB)	[107,109]
Canola	*Wautersia eutropha ATCC 17699*	10.96	P(3HB)	[132]
Cooking	*P.aeruginosa 42A2*	0.76	P(3HB)	[110]
Palm	*C. necator H16*	4.2	P(3HB)	[108]
Rapeseed	*K. xylinus DSM 46602*	6	BC	[111]
Waste water				
Fruit processing	*Halomonas i4786*	1.8	P(3HB)	[133]
Alcohol distillery	*K. saccharivorans* BC1	0.155	BC	[134]
Rice wine distillery	*G. xilynus* BCRC12334	1	BC	[135]
Lipid fermentation	*G. xylinus* CH001	0.1	BC	[136]
Hot water wood sugar extraction	*A. xylinus* 23769	0.019	BC	[137]
Butanol fermentation	*G. xylinus* CH001	0.17	BC	[138]
Jujube	*G. xylinys* CGMC2955	0.375	BC	[139]
WW anaerobically fermented to VFAs				
Municipal	Activated sludge	1.37	P(3HB-*co*-3HV)	[140]
Paperboard mill	Activated sludge	3	P(3HB-*co*-3HV)	[141]
Candy factory f	*Plasticicumulans acidivorans*	0.05 (gPHA/gVSS)	P(3HB-*co*-3HV)	[142]
Urban waste	Activated sludge	0.65 (gPHA/gVSS)	P(3HB-*co*-3HV)	[143]
Sewage sludge and municipal	Activated sludge	8.64	scl-PHA	[144]

**Table 5:** Examples of chemical modifications of bacterial polymers.

	Blend Composition	Key Features	Ref.
PHA	P(3HB)/P(3HB-*co-*3HHx)	Better cell biocompatibility on blend polymer scaffolds of PHBHHx/PHB. PHB crystallization degree decreased with increasing PHBHHx content.	[227,228]
	P(3HB)/P(3HO-*co-*3HHx)	Higher Young’s modulus, tensile strength, thermal stability, tailorable biodegradability, and improved biocompatibility with HMEC-1 cells when compared with P(3HO-*co-3*HHx) films.	[229]
	P(3HB)/lignin	Lignin contents ≤30 wt % reduce the crystallinity of PHB. At higher lignin contents, the blends have higher dynamic storage and loss modulus than pure PHB.	[230]
	P(3HB-*co-*3HV)/PLA	Blends were immiscible for all compositions. Improved thermal stability and significant ductile plastic deformation.	[231]
	mcl-PHA/PLA	Improved elongation at break, lower crystallization, and higher biocompatibility.	[232]
	P(3HB-*co-*3HHx)/polycaprolactone (PCL)	Improved degradation and mechanical and biocompatibility properties.	[233]
BC	BC/poly(methylmethacrylate) (PMMA)	Improved mechanical properties and biocompatibility.	[234]
	BC/antimicrobial PHA (PHACOS)	Antibacterial activity against *S. aureus.*	[235]
γ-PGA	γ-PGA/chitosan (CS)	Improved hydrophilic, cytocompatibility, and mechanical properties.	[236]
	PGA/gelatin	Gelatin stabilizes PGA molecules. Improved mechanical properties and biocompatibility with vascular cells.	[237]
	PCL/PGA	Hydrophilicity and water uptake of the nanofibrous scaffolds increased with PGA content.	[238]
**Polymer**	**Grafting Molecule**	**Key Features**	**Ref.**
P(3HO-*co-*3HU)	Thiolation with Jeffamine^®^	Water-soluble amphiphilic copolymers with thermoresponsive behavior.	[239]
unsaturated PHA	Epoxidation	Epoxidation sped up the crosslinking reaction and resulted in a strong, tear-resistant film with increased tensile strength and Young’s modulus.	[240]
P(3HO), P(3HB)	Chlorination	Increased *Tg* and *Tm*, while the same procedure on P(3HB) translates to a decrease in *Tm* and increase in *Tg*. Modulation of the hydrophobicity of the polymers.	[78]
P(3HB)	Alkali treatment with NaOH	Treatment of P(3HB) surfaces with NaOH enhanced proliferation of human osteoblasts and inhibited *S. aureus* growth	[241]
PHB	PVA	Decreased crystallinity and enhanced biodegradability of the final polymer.	[242]
unsaturated PHA	PNIPAm oligomers	Improved surface hydrophilicity and thermoresponsive properties. Good biocompatibility for cell growth and thermoresponsive cell detachment ability.	[243]
PHA	Fibronectin active fragment (GRGDS peptide)	Exhibited cell adhesiveness and improved biocompatibility.	[244]
P(3HB-*co*-3HV)	RGD-containing peptides	Increased hydrophilicity of the surface of the film and improved cellular compatibility.	[245]
P(3HO)	Vinyl imidazole	Increased hydrophilicity and biocompatibility and showed antibacterial activity against *E. coli* and *S. aureus.*	[246]
P(3HB)	Different amino compounds	Amino-PHB polymers showed antibacterial, antioxidant, and anticancer activities. PHB-ethylendiamine displayed better growth-inhibitory antibacterial activity against *S. aureus, K. pneumoniae, P. aeruginosa,* and *E. coli*. PHB-piperazine showed a potent anticancer effect against in vivo Ehrlich ascitic carcinoma-bearing mice.	[247]
P(3HB), P(3HB-*co*-3HV)	CS and CS oligosaccharides	Decreased thermal stability of the chitosan backbone.	[248]
P(3HO), P(3HB-*co*-3HV)	CS	Solubilization of chitosan-g-PHA graft depends on grafting percentage.	[249]
P(3HB)	Ar plasma	Increased surface polarity; improved cell adhesion, proliferation, and spreading homogeneity on the PHB surface	[250]
P(3HB)	O_2_ plasma	Enhanced hydrophilicity. Ability to directly immobilize T4 bacteriophages, resulting in an antimicrobial material against *E. coli.*	[251]
BC	Aminoalkyl groups	Improved mechanical and thermal properties. Antimicrobial properties against *S. aureus* and *E. coli* and was nontoxic to human adipose-derived mesenchymal stem cells.	[252]
	Oligo peptides, glycyl-L-glutamine or glycyl-glycyl-glycine	Enhanced its interfacial wettability, boosted mineralization induction, and improved affinity between polymeric and mineral phases.	[253]
	RGDC peptides and gentamicin	Growth inhibition of *S. mutans* and promoted fibroblast adhesion and proliferation.	[254]
	Amoxicillin (AM)	Good porosity and swelling behaviors. Antibacterial activities against *E. coli*, *C. albicans*, and *S. aureus* and nontoxic to HEK293 cells	[255]
	AM	Good stability with a slight reduction in swelling capabilities. pH responsiveness with an increase in drug swelling and release at higher pH.	[256]
	CS	Better uniformity of nanosized fibrils, with better acid and temperature stability. Enhanced BC dispersion.	[257]
	N_2_ plasma	Increased porosity and the number of functional groups on the surface of BC, which improved the cell adhesion.	[258]
	O_2_ and N_2_ plasmas	Increased surface hydrophilicity due to the incorporation of carbon–oxygen and amide and amino groups.	[259]
	CF_4_ plasma	Surface presented hydrophobic properties and potential to promote cell adhesion and proliferation due to greater adsorption of proteins on the BC surface.	[259]
PGA	PCL	Improved shear elasticity and compressive strength. Provided effective protection for femoral condyle and tibial plateau cartilage when applied in a rabbit model and regenerated a meniscus-like tissue.	[260]
	Benzyl groups	Novel electrospun biopolymer exhibited rapid shrinkage upon induction by heat or a series of solvents.	[261]
	Phenylalanine (Phe) or leucine (Leu)	Production of bionanoparticles derived from γ-PGA and phenylalanine ethyl ester, with excellent water dispersibility, 200 nm diameters, and surface chemical functionality of carboxyl groups,	[262]
	β-sheet peptides	Hydrogel stiffness can be controlled by changing the β-sheet peptide graft density, the bulk hydrogel concentration, and the ratio of covalently coupled and free peptides. Additional functionality can be incorporated into this self-healing hybrid hydrogel.	[263]
**Material**	**Cross-Linking Agent**	**Key Features**	**Ref.**
BC BC-CS membranes	Tripolyphosphate (TPP)	Optimum water vapor permeability. BC-CS exhibited local and peripheral inhibition of bacterial growth. The presence of ciprofloxacin effectively produced a stronger inhibition effect on both tested bacteria, *P. aeruginosa* and *S. aureus*	[264]
BC/CS blends	Glutaraldehyde	Showed flexibility, high thermal stability, and high mechanical properties. Showed antibacterial properties against tested Gram-positive and Gram-negative bacteria.	[265]
Functionalization of BC with ε-poly-L-Lysine (ε-PLL)	Carbodiimide chemistry	Preserved the good structural and mechanical properties of BC. Inhibited growth of *S. epidermidis* on the membranes and was cytocompatible with human fibroblasts.	[266]
Unsaturated copolyester (PHBU)	Thiol-ene click chemistry	Enhanced tensile strength without affecting cytotoxicity and biocompatibility towards human mesenchymal stem cells.	[267]
Unsaturated PHA copolymer poly[(*R*)-3-hydroxyundecanoate-*co*-(*R*)-3-hydroxy-10-undecenoate] P(HU10U)	Polyethylene glycol dithiol (PDT)	Swelling behavior in different solvents; mechanical and morphological properties could be tuned by varying the ratio of P(HU10U) to PDT. Good biocompatibility.	[268]
PGA-conjugated cysteamine (PGA-SH) and methacrylate-PGA (PGA-GMA)	Michael-addition reactions	Mechanical properties, porous structure, swelling, and degradation process of the hydrogels could be controlled by adjusting modified PGA polymer component. Exhibited good biocompatibility and high stability. Promoted chondrogenesis of loaded BMSCs and facilitated cartilage reconstruction in the defected area in a rabbit auricular cartilage defect model.	[157]
Hydrogels of PGA	N,N,N-trimethyl-3-[(2-methylacryloyl)amino]propan-1-aminium (METH)	Maintained a stable form during the nine weeks of the study. Useful for preparing particles.	[269]
**Cured Material**		**Key Features**	**Ref.**
BC crosslinked with citric acid		Improved rehydration capacity; showed higher porosity, wettability, and water swelling.	[270]
PHA combined with segments of polyurethane (PHP), telechelic-hydroxylated polyhydroxyalkanoate (PHA-diols), and polyethylene glycol (PEG)		Good shape–memory effect (SME) and rapid recovery. Possess thermo- and water-responsive properties, properties of triggering shape-morphing, enabling self-folding and self-expansion of shapes into three-dimensional (3D) scaffolds.	[271]
Light cured methacrylated PGA nanoparticle-created hydrogel system (PGA nanogel)		Good swelling and mechanical properties. Good antibiotic release behavior. Good biocompatibility.	[272]

## Data Availability

Not applicable.
